# Absolute Copy Numbers of β-Actin Proteins Collected from 10,000 Single Cells

**DOI:** 10.3390/mi9050254

**Published:** 2018-05-22

**Authors:** Beiyuan Fan, Xiufeng Li, Lixing Liu, Deyong Chen, Shanshan Cao, Dong Men, Junbo Wang, Jian Chen

**Affiliations:** 1 State Key Laboratory of Transducer Technology, Institute of Electronics, Chinese Academy of Sciences, Beijing 100190, China; fanbeiyuan@ucas.ac.cn (B.F.); lixiufeng13@mails.ucas.ac.cn (X.L.); liulixing16@mails.ucas.ac.cn (L.L.); dychen@mail.ie.ac.cn (D.C.); 2 University of Chinese Academy of Sciences, Beijing 100049, China; 3 State Key Laboratory of Virology, Wuhan Institute of Virology, Chinese Academy of Sciences, Wuhan 430071, China; cao@hotmails.com (S.C.); d.men@wh.iov.cn (D.M.)

**Keywords:** microfluidics, single-cell analysis, polymeric microfluidic flow cytometry, single-cell protein quantification

## Abstract

Semi-quantitative studies have located varied expressions of β-actin proteins at the population level, questioning their roles as internal controls in western blots, while the absolute copy numbers of β-actins at the single-cell level are missing. In this study, a polymeric microfluidic flow cytometry was used for single-cell analysis, and the absolute copy numbers of single-cell β-actin proteins were quantified as 9.9 ± 4.6 × 10^5^, 6.8 ± 4.0 × 10^5^ and 11.0 ± 5.5 × 10^5^ per cell for A549 (n_cell_ = 14,754), Hep G2 (n_cell_ = 36,949), and HeLa (n_cell_ = 24,383), respectively. High coefficients of variation (~50%) and high quartile coefficients of dispersion (~30%) were located, indicating significant variations of β-actin proteins within the same cell type. Low *p* values (≪0.01) and high classification rates based on neural network (~70%) were quantified among A549, Hep G2 and HeLa cells, suggesting expression differences of β-actin proteins among three cell types. In summary, the results reported here indicate significant variations of β-actin proteins within the same cell type from cell to cell, and significant expression differences of β-actin proteins among different cell types, strongly questioning the properties of using β-actin proteins as internal controls in western blots.

## 1. Introduction

As housekeeping proteins, β-actins are obligatory parts of cell cytoskeletons, playing important roles in the maintenance of cellular shapes, migrations, and signal transductions [1]. Due to their constitutive expressions, β-actin proteins are commonly used as internal controls in western blots, based on the assumptions of constant expressions from cell to cell and sample to sample. However, recent studies indicate varied expressions of β-actin proteins; thus, the use of β-actin proteins as internal controls is under question [2,3].

As pioneering studies, in 2005, Banks et al. reported varied expressions of β-actin proteins with (1) coefficients of variation of 28% among 10 renal cancer cell lines; (2) higher levels in tumor versus normal renal tissues; and (3) 4-fold differences between stomach and adrenal tissues [4]. Furthermore, in 2006, Liu et al. reported a 2.5-fold increase in β-actin proteins in injured spinal cords in comparison to normal counterparts [5]. In addition, in 2008, significant differences in β-actin proteins were observed in skeletal muscle tissues of early symptomatic, symptomatic, and terminal stages [6].

More recently, in 2014, Gupta et al. reported higher levels of β-actin proteins in gastric tumor tissues in comparison to normal counterparts [7]. Also in 2014, Deybboe et al. reported decreases in β-actin proteins in human skeletal muscles with aging [8]. Furthermore, in 2015, Nam et al. reported that β-actin proteins were dramatically lower in the proximal duodenum relative to the rest of the small intestines [9]. In addition, in 2016, Chen et al. reported differences of β-actin proteins in the submandibular glands of male and female mice [10].

All of these previous data about the expressions of β-actin proteins were obtained from western blots. As a semi-quantitative approach, it cannot report absolute copy numbers of β-actin proteins, and thus, data reported by different groups cannot be effectively compared with each other. In addition, the previously reported data were derived from population studies, which cannot be used to address questions of whether there exist different expressions of β-actin proteins from cell to cell even within the same cell type.

In order to address this issue, in this study, absolute copy numbers of β-actin proteins were obtained, leveraging a recently reported polymeric microfluidic flow cytometry [11]. More specifically, lung, liver, and cervical tumour cell lines of A549, Hep G2 and HeLa were characterized by the microfluidic platform, yielding absolute copy numbers of β-actin proteins from ~10,000 single cells. Varied expressions of β-actin proteins among individual cells within the same cell type and among different cell types were located. The data reported here may be used as references for future studies of β-actin proteins.

## 2. Materials and Methods

### 2.1. Materials

All cell-culture reagents were purchased from Life Technologies Corporation (Grand Island, NY, USA). Materials required for device fabrication included SU-8 photoresist (MicroChem Corporation, Westborough, MA, USA) and polydimethylsiloxane (PDMS, 184 silicone elastomer, Dow Corning Corporation, Midland, MI, USA).

More specifically, materials in cell culture and staining include RPMI-1640 medium (GIBICO, Life Technologies Corporation, Grand Island, NY, USA), DMEM medium (GIBICO, Life Technologies Corporation, Grand Island, NY, USA), fetal bovine serum (GIBICO, Life Technologies Corporation, Grand Island, NY, USA), penicillin and streptomycin (GIBICO, Life Technologies Corporation, Grand Island, NY, USA), trypsin (GIBICO, Life Technologies Corporation, Grand Island, NY, USA), phosphate buffer saline (GIBICO, Life Technologies Corporation, Grand Island, NY, USA), FITC labelled anti-β-actin antibody (ABCAM, ABCAM Corporation, Cambridge, UK), paraformaldehyde (Sigma, Sigma-Aldrich Corporation, St. Louis, MO, USA), triton x-100 (Sigma, Sigma-Aldrich Corporation, St. Louis, MO, USA), and bovine serum albumin (Sigma, Sigma-Aldrich Corporation, St. Louis, MO, USA).

### 2.2. Working Flowchart

**Figure 1:**
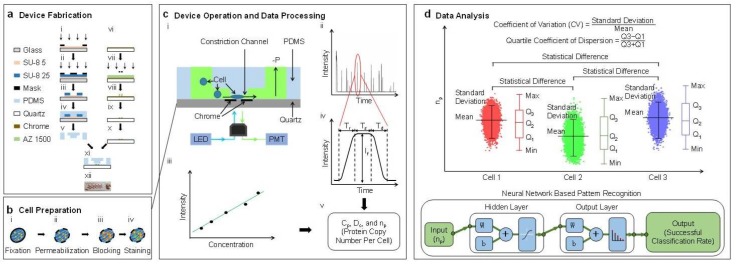
Methodology. Working flowchart for the characterization of the absolute copy number of β-actin proteins at the single-cell level. Key steps include device fabrication (**a**); cell preparation (**b**); device operation & data processing (**c**) and data analysis (**d**). In this study, single cells stained with fluorescence labelled antibodies are forced to deform through a polymeric constriction channel (microfabricated channel with a cross-sectional area smaller than a cell) where the obtained fluorescent profiles are translated to cellular sizes and absolute copy numbers of specific intracellular proteins. Coefficients of variation and quartile coefficients of dispersion were quantified to determine the varied expressions of β-actin proteins among individual cells within the same cell. Statistical +analysis and neural network based pattern recognition were conducted to determine the varied expressions of β-actin proteins among different cell types.

The characterization of the absolute copy numbers of single-cell β-actin proteins mainly includes four steps: device fabrication, cell preparation, device operation & data processing, and data analysis (see Figure 1). In this study, single cells stained with fluorescence labeled antibodies are forced to deform through a polymeric constriction channel (microfabricated channel with a cross-sectional area smaller than a cell) where fluorescent profiles are collected as a function of time, which are further translated to cellular sizes and absolute copy numbers of specific intracellular proteins. Coefficients of variation and quartile coefficients of dispersion were quantified to determine the varied expressions of β-actin proteins among individual cells within the same cell. Statistical analysis and neural network based pattern recognition were conducted to determine the varied expressions of β-actin proteins among different cell types.

### 2.3. Device Design and Fabrication

In this study, a constriction channel with a cross-sectional area of 8 μm × 8 μm and a chrome gap of 2.5 μm in width was chosen for single-cell protein characterization [11]. The cross-sectional area of 8 μm × 8 μm ensures that cells with a mean diameter of 15 μm deform through and fully fill the constriction channel. In order to divide fluorescent pulses of traveling cells into rising domains, stable domains and declining domains, the gap of the chrome window should be as small as possible; 2.5 μm was used in this study.

As shown in Figure 1a, the proposed device was fabricated based on conventional microfabrication, including key steps of SU-8 mould fabrication (see Figure 1(a-i)–(a-iii)), PDMS replication (see Figure 1(a-iv),(a-v)), chrome layer patterning (see Figure 1(a-vi)–(a-x)), and bonding (see Figure 1(a-xi),(a-xii)). Detailed fabrication steps can be found from [11].

### 2.4. Cell Preparation

All cell lines were purchased from China Infrastructure of Cell Line Resources and cultured in a cell incubator (3111, Thermo Scientific, Waltham, MA, USA) at 37 °C in 5% CO_2_. More specifically, a lung tumor cell line of A549, a liver tumor cell line of Hep G2, and a cervical tumor cell line of HeLa were cultured with RPMI-1640, DMEM and DMEM media, respectively, which were supplemented with 10% Fetal Bovine Serum (FBS) and 1% penicillin and streptomycin. Prior to experiments, cells were trypsinized, centrifuged, and resuspended in phosphate buffer saline with 0.5% bovine serum albumin at a concentration of ~1 million cells per mL.

Intracellular staining of β-actin proteins was conducted, following well-established protocols used in flow cytometry [12,13], which included key steps of fixation (see Figure 1(b-i)), membrane permeabilization (see Figure 1(b-ii)), blocking (see Figure 1(b-iii)), and antibody staining (see Figure 1(b-iv)). Firstly, the cell suspension was mixed with a 2% formaldehyde solution and incubated for 15 min at 4 °C for fixation. Then, triton x-100 (0.05% for A549 cells, 0.03% for Hep G2 cells, and 0.1% for HeLa cells) was added for an incubation of 15 min at 4 °C, in order to penetrate cellular membranes. Then blocking was conducted based on 5% vs. 1% bovine serum albumin for 30 min at room temperature. Both FITC labelled anti-β-actin antibodies (1:100) and isotype controls (1:34.5 the same final concentration as anti-beta actin antibody) were used to stain cells in suspension for 1, 2, 4, or 8 h at 37 °C for comparison. After the step of staining, cells were divided into two portions which were placed on an inverted fluorescence microscope (IX 83, Olympus, Tokyo, Japan) for imaging, and applied to the microfluidic constriction channel for fluorescent detections, respectively.

### 2.5. Device Operation and Data Processing

In operations, the microfluidic constriction channel was first filled with phosphate buffer saline with 0.5% bovine serum albumin. Then, suspended cells stained with FITC labelled anti-β-actin antibodies or isotype controls were applied to the entrance of the cell loading channel where a negative pressure of roughly 10 kPa generated from a pressure calibrator (DIP-610 pressure calibrator, Druck, UK) was used to aspirate cells continuously through the constriction channel (see Figure 1(c-i)). Fluorescence of single cells travelling in the constriction channel was captured by a photomultiplier tube (PMT, H10722-01, Hamamatsu, Japan), and sampled by a data acquisition card (PCI-6221, National Instruments, Austin, TX, USA) at a sampling rate of 100 kHz (see Figure 1(c-ii)). In calibrations, solutions with FITC labelled anti-β-actin antibodies were applied into the constriction channel under the same conditions as experiments (see Figure 1(c-iii)).

The fluorescent pulse of a representative cell was divided into three domains: a rising domain with a time duration of T_r_, a stable domain with a fluorescent level of I_f_ and a time duration of T_s_, and a declining domain with a time duration of T_d_ (see Figure 1(c-iv)). These raw parameters were then translated to the diameters of cells (D_c_), concentration of β-actins at the single-cell level (C_p_), and the absolute copy number of β-actins (n_p_) (see Figure 1(c-v)) [11].

### 2.6. Data Analysis

The measurement results of the absolute copy numbers of single-cell β-actin proteins of the same cell type were represented as means ± standard deviations with three quantified quartiles (e.g., Q_1_, Q_2_ and Q_3_). Dimensionless parameters, including the coefficients of variation (the ratio of the standard deviation to the mean) and the quartile coefficient of dispersion (Q_3_ − Q_1_)/(Q_3_ + Q_1_)), were calculated to evaluate the expression differences of β-actin proteins from cell to cell within the same cell type.

In addition, analysis of variance (ANOVA) was used to locate statistical differences of β-actin proteins among A549, Hep G2, and HeLa cells, where values of *p* < 0.01 (*) were considered as statistically significant. Furthermore, neural network based pattern recognitions were conducted based on a ‘Neural Network Pattern Recognition App’ (MATLAB 2010, MathWorks, Natick, MA, USA) to differentiate the distribution of β-actin proteins among these three cell types. The app employs a two-layer (hidden and output layer) feed forward neural network, with sigmoid hidden and softmax output neurons [14,15].

## 3. Results

**Figure 2:**
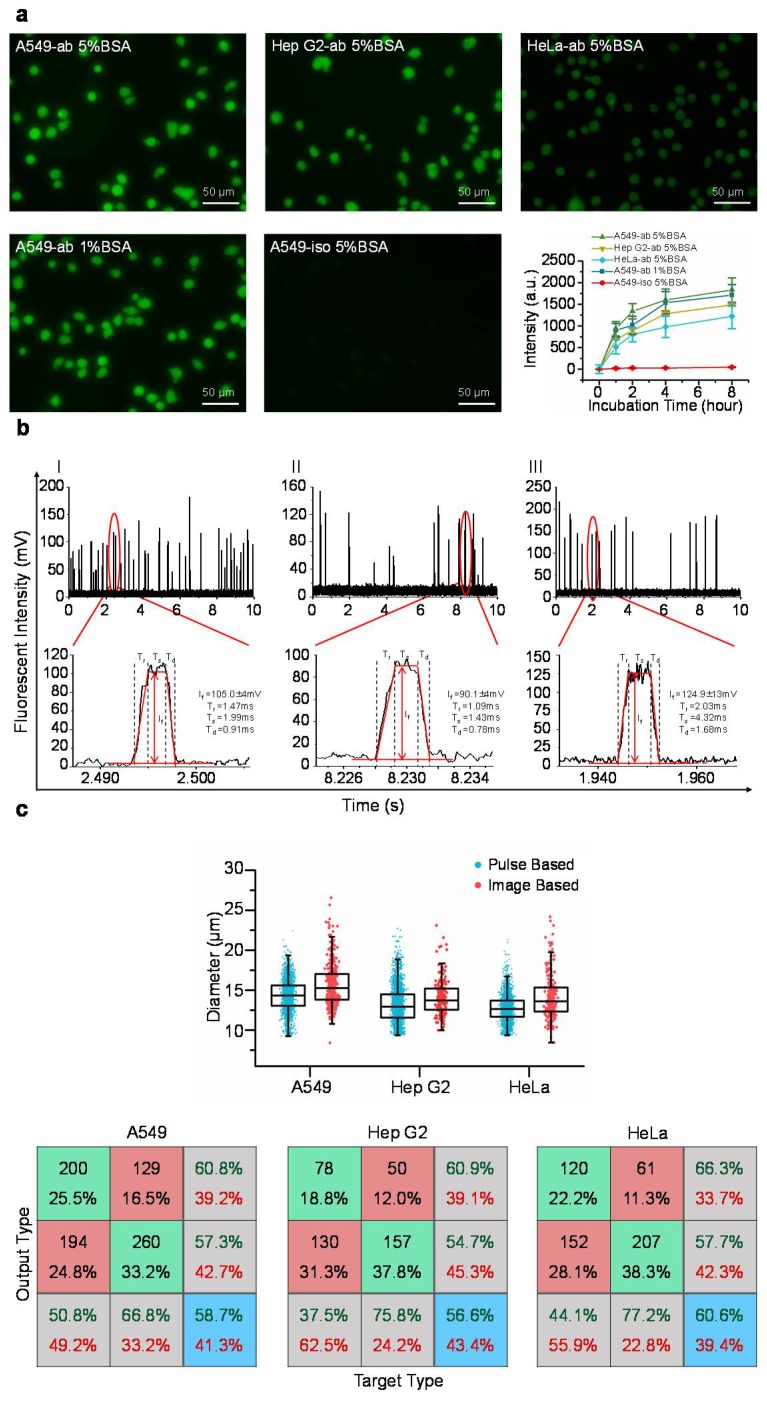
(**a**) Fluorescent pictures of stained A549, Hep G2, and HeLa cells where the intensities of single cells stained with fluorescence labelled anti-β-actin antibodies or isotype controls were quantified as a function of time under two concentrations of bovine serum albumin (1% vs. 5%) for blocking. These results validated the process of intracellular staining where (1) all the exposed proteins are taken by the fluorescence labelled antibodies and (2) non-specific sites within cells are properly blocked; (**b**) Fluorescent pulses of travelling A549 (I), Hep G2 (II), and HeLa (III) cells can be effectively divided into rising domains, stable domains and declining domains based on curve fitting; (**c**) The scatter plots of diameters of cells based on the processing of fluorescent pulses vs. images of microscopy where neural network based pattern recognition produced successful classification rates of 58.7% of A549 cells, 56.6% of Hep G2 cells and 60.6% of HeLa cells. These results indicate that comparable cell diameters were obtained based on curve fitting of fluorescent pulses and processing of microscopic images, validating the processing of fluorescent pulses.

Figure 2a shows representative fluorescent pictures of stained A549, Hep G2, and HeLa cells where the intensities of single cells stained with fluorescence labelled anti-β-actin antibodies or isotype controls were quantified as a function of time. It was observed that the intensities of stained single cells initially increased with the incubation time, and then showed the signs of saturation at 4 h. Further increases in the incubation time (e.g., eight hours) did not lead to further significant increases in the fluorescent intensities, suggesting that after four hours of incubating cells with fluorescence labelled antibodies, all the intracellular β-actin proteins were bound with fluorescence labelled antibodies.

In addition, two blocking parameters of 1% and 5% bovine serum albumin solutions produced comparable fluorescent intensities, indicating that non-specific intracellular sites were properly occupied by bovine serum albumin, and thus, the issue of non-specific binding is not a concern (see Figure 2a). Furthermore, the intensities of isotype controls were two orders lower than the intensities obtained from fluorescence labelled antibodies, further addressing the potential concern of non-specific binding in the step of intracellular staining (see Figure 2a).

Figure 2b shows the preliminary measurement results of travelling A549, Hep G2, and HeLa cells with corresponding pulses effectively divided into rising domains, stable domains and declining domains. By processing these raw parameters, the diameters of cells (D_c_) were quantified as 14.3 ± 1.9 μm (A549, n_cell_ = 14,754), 13.1 ± 2.2 μm (Hep G2, n_cell_ = 36,949), and 12.7 ± 1.6 μm (HeLa, n_cell_ = 24,383). These results were consistent with the diameters of cells (D_c_) of 15.7 ± 2.6 μm (A549, n_cell_ = 394), 13.9 ± 2.5 μm (Hep G2, n_cell_ = 195), and 14.1 ± 2.7 μm (HeLa, n_cell_ = 268) obtained from image processing of cell pictures, validating the processing of fluorescent pulses (see Figure 2c and Table 1).

Neural network based pattern recognition produced successful classification rates of 58.7% of A549 cells, 56.6% of Hep G2 cells, and 60.6% of HeLa cells, when two groups of cell diameters were compared (see Figure 2c). These values of successful classification rates are within the range of 55–60%, suggesting comparable diameters obtained from fluorescent pulses and microscopic images, which further confirms the processing of fluorescent pulses.

**Figure 3:**
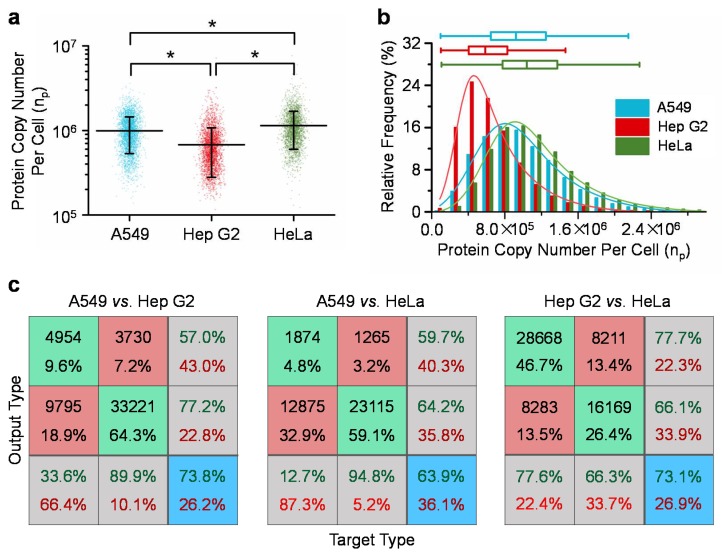
(**a**) Scatter plot of the absolute copy numbers of single-cell β-actin proteins of A549 (n_cell_ = 14,754), Hep G2 (n_cell_ = 36,949) and HeLa (n_cell_ = 24,383) cells with means and standard deviations included (* represents the statistical difference with *p* < 0.01); (**b**) Distributions of absolute copy numbers of β-actin proteins at the single-cell level for A549, Hep G2 and HeLa cells with three quartiles and the quartile coefficients of dispersion included; (**c**) Neural network was used to evaluate the distribution differences of β-actin proteins among A549, Hep G2 and HeLa cells, producing successful classification rates of 73.8% for A549 vs. Hep G2 cells, 63.9% for A549 vs. HeLa cells and 73.1% for Hep G2 vs. HeLa cells.

Figure 3 summarizes the quantified single-cell copy numbers of β-actins of A549, Hep G2, and HeLa cells. For A549 (n_cell_ = 14,754), Hep G2 (n_cell_ = 36,949) and HeLa (n_cell_ = 24,383) cells, absolute copy numbers of beta-actins were quantified as 9.9 ± 4.6 × 10^5^, 6.8 ± 4.0 × 10^5^ and 11.0 ± 5.5 × 10^5^ per cell, respectively. The coefficients of variation were quantified as 46.4% for A549, 58.9% for Hep G2, and 47.8% for HeLa cells, which significantly deviated from 0%, and indicated significant variations of β-actin proteins from cell to cell for A549, Hep G2 and HeLa cells, respectively (see Figure 3a and Table 1). Furthermore, three quartiles and the quartile coefficients of dispersion were quantified as 6.53 × 10^5^, 9.27 × 10^5^, 1.25 × 10^6^, and 31.5% for A549 cells, 4.11 × 10^5^, 5.92 × 10^5^, 8.34 × 10^5^, and 33.9% for Hep G2 cells and 7.81 × 10^5^, 1.05 × 10^6^, 1.38 × 10^6^, and 27.7% for HeLa cells, respectively. These values of quartile coefficients of dispersion significantly deviated from 0%, further indicating the significant variations of β-actin proteins within the same cell types (see Figure 3b).

As to the comparisons among three cell types (A549, Hep G2 and HeLa), statistical significances were located based on ANOVA, indicating the existences of expression differences of β-actins among these three cell types (see Figure 3a). Neural network based pattern recognition produced successful classification rates of 73.8% for A549 vs. Hep G2 cells, 63.9% for A549 vs. HeLa cells, and 73.1% for Hep G2 vs. HeLa cells (see Figure 3c). These values of successful classification rates are significantly higher than 50% as an indicator of no distribution difference between two cell types, further confirming expression differences of β-actins among these three cell types.

In this study, the absolute copy numbers of single-cell β-actin proteins of A549 cells were compared to the population approaches based on the conventional enzyme-linked immunosorbent assay (ELISA), producing the results at the same order, which were 1.0 ± 0.5 × 10^6^ vs. 3.6 ± 0.2 × 10^6^ per cells, respectively [11]. Actually, intracellular staining in flow cytometry has been functioning as a well-established semi-quantitative approach in deep phenotyping [16,17] and signaling state characterization [18,19,20,21], which has been demonstrated to be capable of producing trustworthy results.

## 4. Conclusions

In this study, the copy numbers of β-actin proteins from ~10,000 single cells were reported, based on a previously developed microfluidic platform, and the results were validated by the quality controls in key steps of experimental operations and data analysis. Based on data analysis, significant variations of β-actin proteins within the same cell type from cell to cell and significant expression differences of β-actin proteins among different cell types were located, strongly questioning the use of β-actin proteins as internal controls in western blots, with the assumption of constant expressions of β-actin proteins from cell to cell and sample to sample.

## Figures and Tables

**Table 1:** A summary of quantified key parameters of A549, Hep G2 and HeLa cells including T_r_ (time duration of the rising domain for a fluorescent pulse representing a traveling cell), T_s_ (time duration of the stable domain for a fluorescent pulse representing a traveling cell), T_d_ (time duration of the declining domain for a fluorescent pulse representing a traveling cell), I_f_ (fluorescent level of the stable domain for a fluorescent pulse representing a traveling cell), D_c_ (diameter of cells), C_p_ (concentration of β-actins at the single-cell level) and n_p_ (absolute copy number of β-actin proteins at the single-cell level).

Cell Type	T_r_ (ms)	T_s_ (ms)	T_d_ (ms)	I_f_ (mv)	D_c_ (μm)	C_p_ (μM)	n_p_ (/cell)
A549 (n_cell_ = 14,754)	2.0 ± 1.6	4.5 ± 4.3	1.5 ± 1.2	85.0 ± 24.4	14.3 ± 1.9	1.0 ± 0.3	9.9 ± 4.6 × 10^5^
Hep G2 (n_cell_ = 36,949)	1.6 ± 2.3	2.9 ± 5.6	1.4 ± 3.0	75.5 ± 26.2	13.1 ± 2.2	0.9 ± 0.3	6.8 ± 4.0 × 10^5^
HeLa (n_cell_ = 24,383)	2.6 ± 2.9	3.9 ± 5.3	1.9 ± 2.2	132.4 ± 34.5	12.8 ± 1.6	1.7 ± 0.5	11.4 ± 5.5 × 10^5^
